# Pixel shift-based reversible steganography for privacy-preserving medical images

**DOI:** 10.3389/fmed.2026.1871854

**Published:** 2026-08-19

**Authors:** Xiaofeng Huang, Bing Zhang, Xishun Zhu

**Affiliations:** 1 School of Mathematics and Statistics, Hainan Normal University, Haikou, China; 2 School of Intelligent Manufacturing, Nanyang Institute of Technology, Nanyang, Henan, China

**Keywords:** ablation study, bit error rate, image hiding, lossless, privacy protection, recurrent codes

## Abstract

Steganography plays a critical role in preserving the privacy of medical images by protecting sensitive patient information without compromising image quality or diagnostic utility. This paper proposes a novel lossless privacy protection algorithm for medical images based on pixel displacement. The core innovation lies in the synergistic combination of physical transformation and deep learning—specifically, the integration of shift-rectification with a dual-carrier embedding mechanism. The proposed method begins by applying byte-level pixel shifting to the confidential medical image to generate a shift-rectified version. A deep convolutional neural network is then employed to embed both the original and the rectified medical images into two separate carrier images, ensuring that the resulting container images are visually indistinguishable from the carriers. Subsequently, a dedicated recovery network extracts and reconstructs the lossy shift-rectified image and a degraded version of the original from the two carriers, using the former to calibrate and fully restore the original medical image in a lossless manner. Experimental results demonstrate the algorithm’s effectiveness in preserving privacy while enabling perfect image recovery: the container images achieve an average PSNR of 40.08 dB and MSSIM of 0.988 relative to the carriers, confirming near-perceptual-indistinguishability; the reconstructed secret images are recovered with a bit-level accuracy of 100%; and the algorithm maintains robustness under Gaussian noise, salt-and-pepper noise, and PCA compression with lossless recovery guaranteed. Moreover, the method shows excellent generalization across multiple medical imaging datasets (CT, MRI, and X-ray), highlighting its clinical applicability and innovation in secure medical image transmission systems.

## 1 Introduction

The privacy protection of medical images has gained increasing importance in recent years, particularly with the widespread adoption of electronic medical records and telemedicine. The storage, transmission, and sharing of medical image data now face significant challenges. To prevent patient privacy from being compromised and to enhance data security, various privacy protection technologies, such as image encryption, homomorphic encryption, and image hiding, are being actively developed (1).

Image encryption is an effective method for safeguarding the privacy and integrity of image data. By employing encryption techniques, only authorized physicians or medical personnel can access a patient’s medical images, thereby protecting sensitive medical information and reducing the risk of privacy breaches (2). The integration of symmetric cryptography with blockchain technology and distributed storage systems ensures the confidentiality of personal health records, verifiability of search results, and forward security of the system, offering practical viability for large-scale datasets. However, the transmission and storage of encryption keys remain vulnerable to potential attacks (3). Abdellatef et al. (4) employed convolutional neural networks to extract facial features, adapted chaotic maps to generate an encryption matrix, and applied chaotic magic transforms along with XOR operations for image scrambling, resulting in an unrecognizable encrypted CT image. Nevertheless, the reconstructed medical images exhibited minor discrepancies.

Chang et al. (5) segmented stereoscopic medical images into upper, middle, and lower regions, designating a portion as a mask-segmented region of interest. They merged the three masked segments and used the composite mask to partition the entire stereoscopic image into background and region of interest (ROI) areas. The ROI was extracted and transformed into a new cube, followed by the design of a cube rotation scrambling diffusion algorithm. Audio data containing patient medical and diagnostic information was then embedded as a watermark within the encrypted ROI.

Owing to its powerful feature extraction and automatic modeling capabilities, deep learning has been increasingly integrated with encryption for medical image privacy protection. Ding et al. (6) utilized a deep learning-based key generation network as a stream cipher generator to produce private keys for encrypting and decrypting medical images. Selvakumar and Lokesh (7) developed a Zigzag Generative Adversarial Network to generate encryption keys, securing medical images using a Zigzag encryption pattern in conjunction with XOR operations. To enhance security, speckle noise was introduced during preprocessing and subsequently removed after decryption to restore the original image, thereby improving the robustness of both encryption and decryption processes. Huang et al. (8) implemented an enhanced SKK image encryption method, providing parameters to balance trade-offs between image security and other operational requirements. Additionally, they applied gradient-weighted class activation mapping (GradCAM) to explore image-adaptive encryption strategies, training deep neural networks to safeguard medical privacy.

Medical images are typically large digital files, and fully encrypting them can be computationally intensive. Alwan et al. (9) proposed a selective and partial encryption approach for medical images. By employing U-Net to locate tumor regions and integrating hyperchaotic systems, the Integer Wavelet Transform, and Feistel network encryption techniques, they significantly enhanced encryption efficiency and reduced processing time. Although encryption improves privacy, it entails trade-offs, particularly in resource-constrained environments or with large datasets, due to high computational demands. Furthermore, encryption may lead to decryption errors.

Homomorphic encryption enables computations to be performed directly on encrypted data without requiring decryption (10), allowing third parties to process data while preserving both privacy and utility. The core principle is that operations on encrypted data yield results equivalent to those obtained from unencrypted data. Xiang et al. (11) developed a medical image fusion model based on convolutional neural networks and the Inception network, integrating it into a blockchain consensus framework. To optimize computational resources, they introduced a mechanism wherein consensus nodes train the model, while privacy is preserved using efficient homomorphic encryption. However, partially homomorphic encryption supports only a single operation, and fully homomorphic encryption remains computationally expensive and slow, making it less practical for real-time medical image processing.

Steganography offers an effective method for medical image privacy protection, particularly in healthcare settings where patient confidentiality is essential. By embedding sensitive data into medical images, steganography secures and conceals the information. For example, Ren et al. (12) applied adaptive interpolation to identify seed pixels and combined this with a bit histogram shifting technique to ensure reversibility of the embedded content. This method preserves sensitive elements, such as electronic medical records and image signatures, while maintaining image fidelity. Bassem (13) employed a three-dimensional chaotic system and quantum walks to drive the particle swarm optimization algorithm, wherein the generated velocity sequence was utilized to substitute the confidential data, and the position sequence was employed to select the embedding locations within the carrier image for hosting the substituted confidential data.

With the rapid advancement of deep learning, particularly the introduction of convolutional neural networks (CNNs) and generative adversarial networks (GANs), image steganography is evolving toward greater security, enhanced dynamism, and higher automation. Unlike conventional techniques that rely on manually crafted embedding strategies, deep learning-based steganographic models can autonomously learn optimal embedding representations, significantly improving anti-detection capability while maintaining high visual quality of the stego images. Zhu et al. (14) constructed a residual CNN (ResCNN) operating in the wavelet domain, discarding low-frequency coefficients and utilizing only high-frequency sub-bands for encoding, thereby effectively enhancing embedding capacity and resistance against steganalytic attacks.

With respect to adversarial training and GAN-based frameworks, Li et al. (15) designed AdvSGAN, which introduces two sets of adversaries—internal and externa—to simulate real-world attack scenarios. A steganographic noise constraint is imposed on the generator to balance minimal image modification with reliable payload retention. Yuan et al. (16) proposed an end-to-end GAN-based steganographic system that combines adversarial attacks with pixel-wise deep fusion, employing the Wasserstein GAN formulation to ensure convergence stability. Their multi-loss optimization strategy substantially enhances visual fidelity and embedding reliability.

In the field of generative steganography, Yang et al. (17) introduced the PARIS framework, which embeds information into latent vectors following a Gaussian distribution, rendering stego images statistically indistinguishable from regular GAN outputs. Wen et al. (18) utilized StyleGAN2 to generate highly practical stego images and devised a transformation mechanism to further fortify imperceptibility and tamper resistance. Su et al. (19) developed StegaStyleGAN, incorporating a distribution-preserving secret data modulator (DP-SDM) and a secret data extractor (SDE); this architecture maintains latent space consistency and enhances generalization under various attacks. Furthermore, Xiao et al. (20) proposed a Transformer-based adversarial framework, in which an attention mechanism is employed during the embedding phase to extract carrier image features, while a WGAN discriminator is leveraged to refine the imperceptibility of the encoder network.

Focusing on the specific scenario of medical images, Saidi et al. (21) utilized Mask R-CNN to localize regions of interest (ROI) and selectively embedded data exclusively in non-ROI regions. Although this approach achieves a relatively high embedding capacity, the recovery process remains lossy. In contrast, the PRDHMCE algorithm proposed by Boateng et al. (22) separates contrast enhancement from data hiding into distinct regions, enabling completely lossless recovery of the original medical images. However, its effectiveness heavily depends on precise ROI segmentation, and the selection strategy of the “stretching point” based on local histogram statistics is sensitive to noise.

Integrating multiple medical image privacy protection techniques has emerged as a research focus, as it combines the strengths of diverse methods to safeguard data without degrading image quality or usability. Shi et al. (23) merged encryption and data hiding by using plaintext encryption for ROIs and embedding encrypted privacy data into non-region of interest (NROI) areas. Cheng et al. (24) encrypted medical images and separately handled patient privacy data, embedding it reversibly into the encrypted images to form marked encrypted shares, thereby achieving distributed storage and enhancing security. Meng et al. (25) fused cryptography with watermarking, embedding encrypted patient data and watermarks into NROI using steganography. Lishomwa and Zimba (26) introduced a system employing AES-128 symmetric key encryption, with Diffie–Hellman key exchange for secure key generation. To minimize distortion, they applied the Least Significant Bit (LSB) technique to embed data within image pixels while preserving image quality. However, the resulting container images may exhibit limited visual quality and robustness.

Despite the notable progress reviewed above, several critical limitations persist—lossy recovery, weak robustness to attacks, static region-agnostic embedding, lack of correlation modeling for extraction, and reliance on perceptual metrics rather than bit-level verification for losslessness claims—which collectively motivate our proposed algorithm.

To address these issues, the proposed method introduces a pixel-shift dual-carrier mechanism for bit-exact lossless recovery, an adaptive embedding strategy aware of local gradient, texture, and flatness, an APC-CAM module that models adjacent pixel correlation to enhance extraction, and a hybrid attention module integrating spatial, channel, and self-attention. Comprehensive robustness evaluations under multiple attacks with BER-based verification are also conducted.

The main contributions of this paper are:

1. A pixel-shift dual-carrier mechanism enabling truly lossless bit-level recovery of medical images.
2. An adaptive hiding strategy with region-aware embedding that preserves container quality and recoverability.
3. An APC-CAM module that improves extraction accuracy by modeling adjacent pixel correlation disruption.
4. A hybrid attention module that optimizes local details and global structure for high-fidelity reconstruction.
5. Comprehensive robustness evaluation under six attack types with BER-based losslessness validation.

The remainder of this paper is organized as follows. Section 2 reviews related work. Section 3 presents the proposed algorithm. Section 4 describes the experimental setup. Section 5 reports results and comparisons. Section 6 concludes the paper.

## 2 Related work

### 2.1 Pixel shifting in the secret medical image

**Figure 1:**
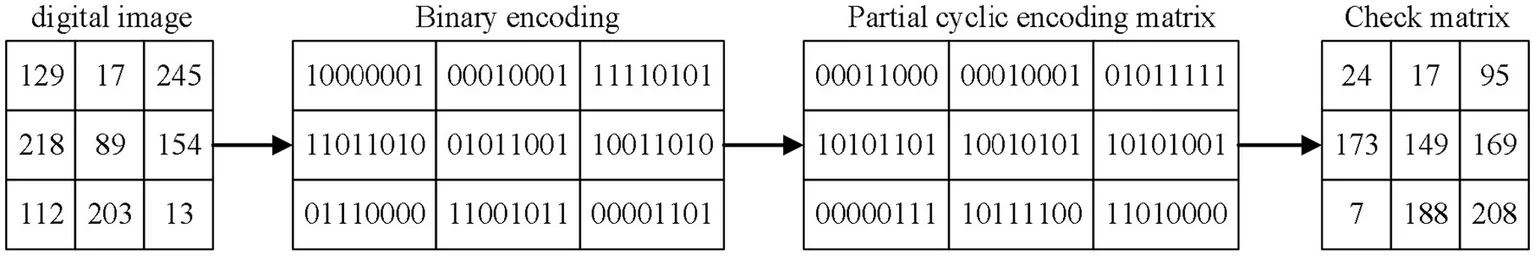
Encoding of the secret image.

The pixel values in a digital image typically range from 0 to 255. As illustrated in Figure 1, after converting the image to binary format, each pixel value is represented using 8 bits. By exchanging the first four bits with the last four bits, a new image, which is referred to as the shift-rectify image, is produced. This image serves to rectify the secret image recovered at the receiving end, ensuring accurate and lossless restoration of the embedded information.

### 2.2 Adaptive hiding strategy

Convolutional neural networks extract feature maps across multiple layers during the convolutional process. These feature maps capture not only fundamental textures, edges, and color attributes of both the container and secret images, but also higher-level latent semantic information. By effectively integrating these feature representations, steganographic information can be embedded while preserving the structural integrity and visual quality of the container image.

To facilitate effective information embedding, the feature maps extracted from the convolutional layers are fused using an adaptive mechanism based on weighted superposition. This approach enables dynamic adjustment of the steganographic embedding process according to the characteristics of the carrier image. The convolutional feature maps typically represent various levels of information, ranging from low-level features, such as edges and textures, to high-level semantic attributes. Analyzing these maps allows identification of regions more suitable for embedding additional steganographic data and regions that should be avoided or carry limited information. The objective of the adaptive weighting mechanism is to quantify the “importance” or “information-carrying capacity” of each region and adjust the embedding strategy accordingly.

Adaptive hiding strategy process: Assume the feature maps extracted by the CNN are denoted as, where C represents the number of channels, H is the height, and W is the width of the feature maps. Each pixel’s features can encompass various types of information, such as color, texture, and edges.

Image gradient information represents the changes or edges within an image region, and is defined as follows:


$$\|\text{Grad}\|=\sqrt{{\left({\text{Grad}}_{x}\right)}^{2}+{\left({\text{Grad}}_{y}\right)}^{2}}=\sqrt{{\left(\frac{\partial F}{\partial x}\right)}^{2}+{\left(\frac{\partial F}{\partial y}\right)}^{2}}$$ (1)

Assume the feature maps extracted by the CNN are denoted as $F\in R^{C\times H\times W}$ , where *C* represents the number of channels, *H* is the height, and *W* is the width of the feature maps. Each pixel’s features can encompass various types of information, such as color, texture, and edges.

1. Calculate the information on the image texture, edges, and changes of the features.

- Image gradient information represents the changes or edges within an image region.

‖Grad‖ represents the degree of the gradient change in this region, ${\text{Grad}}_{x}{\text{Grad}}_{y}$ represent the gradients in the horizontal and vertical directions, respectively.

- The texture information of an image is calculated using convolution operations to determine the variance in local regions:

$$\mathrm{Var}(F)=\frac{1}{k^{2}}\sum_{i,j}{\left(F(i,j)-\mu \right)}^{2}$$

where, μ is the mean of the local region, and $k^{2}$ is the size of the local region.

- The flatness of an image can be measured by calculating the mean and variance of local regions. If the variance is small, it indicates that the region is flat. For a given local region *R* (typically a window of size w×w ), the mean and variance of the region are computed and used to assess the flatness of the area.

The mean value of the local regions is defined as:


$$\mu_{R}=\frac{1}{w^{2}}\sum_{(i,j)\in R}F(i,j)$$

there *R* is the local region, w×w is the size of the region, and (*i*, *j*) represents each pixel within the region.

The variance of the local regions is defined as:


$$\sigma_{R}^{2}=\frac{1}{w^{2}}\sum_{(i,j)\in R}{\left(F(i,j)-\mu_{R}\right)}^{2}$$

There $\mu_{R}$ is the mean value of this region, F(i,j) is the value of the pixels in the region, $\sigma_{R}^{2}$ is the variance.

Image flatness $F_{R}$ can be represented by the variance in the local regions:


$$F_{R}=\sigma_{R}^{2}$$

1. Calculate embedding weights

After obtaining the local features, adaptive weights are calculated based on these features. These features are mapped to the weight space by learning a nonlinear function.

- Combine the gradient information, texture information, and flatness of the image into a comprehensive metric. This be achieved through a weighted sum:

$${\text{feature}}_{i,j}=\alpha_{1}\;{\left\|\text{Grad}(F)\right\|}_{i,j}+\alpha_{2}\mathrm{Var}{\left(F\right)}_{i,j}+\alpha_{3}{\left(F_{R}\right)}_{i,j}$$

where $\alpha_{1},\alpha_{2},\alpha_{3}$ is weight coefficient used to adjust the importance of each feature.

- Using the obtained comprehensive feature value ${\text{feature}}_{i,j}$ , calculate the embedding weight through a nonlinear mapping function Sigmoid:

$$W_{i,j}=\text{Sigmoid}(F_{w}\times {\text{feature}}_{i,j})$$

Where $F_{w}$ is the learned weight parameters, $W_{i,j}$ represents the embedding weight for the region, the range is [0, 1].

- Generate an adaptive embedding weight map. By applying the above operations to the entire feature map, an adaptive weight map $W\in R^{H\times W}$ is obtained, where the value $W_{i,j}$ of each pixel represents the embedding capacity at that location.

1. Deep fusion and information embedding

Where F is features extracted from the carrier image, W is the resulting adaptive weights were calculated, S is the image features that need to be embedded.


$$F^{*}=F⊕(S⊗W)$$

### 2.3 Convolutional attention mechanism based on the correlations of adjacent pixels (APC-CAM)

During the process of embedding hidden information into an image, pixel values are often randomly modified or complex algorithms are applied. These subtle adjustments reduce the correlation between adjacent pixels, especially in initially smooth regions. This random perturbation of pixel values disrupts the natural correlation between adjacent pixels. Based on this, an attention mechanism can be constructed using the correlation between adjacent pixels to enhance the detection and extraction of hidden information. By dynamically adjusting the attention mechanism to focus on changes in adjacent pixels, it becomes possible to more effectively capture the features of the hidden information, thereby improving the accuracy of steganographic information extraction.

The attention mechanism based on adjacent pixel correlation can calculate the “attention weight” for each pixel region by modeling the relationships between adjacent pixels in the image. This weight determines the level of attention given to the region during the extraction of steganographic information.

1. Constructing adjacent pixel correlation

Based on gradient information, an adjacent pixel correlation map is created.


$$A_{i,j}=e^{-\lambda {\text{Grad}}_{i,j}}$$

${\text{Grad}}_{i,j}$ represents the gradient at position (*i*, *j*), which is calculated using Equation 1.

1. Calculation of the convolution of attention weights.

Based on the adjacent pixel correlation map $A_{i,j}$ , the degree of attention of each pixel in the image is dynamically adjusted based on the convolutional attention mechanism.

- Calculate the attention weights.

Based on the adjacent pixel correlation map $A_{i,j}$ , an attention weight is computed for each pixel in the image. First, the adjacent pixel correlation map $A_{i,j}$ is passed through a convolutional layer to generate the attention map α :


α=Conv(A,K)

where Conv(A,K) represents the convolution operation, *K* is the convolution kernel, and $\alpha \in R^{H\times W}$ is the attention map generated through the convolution.

- Normalized attentional weights.

To ensure that the attention weight of each pixel is within [0,1], Softmax operates on the attention weight:


$$\alpha^{*}=\text{Softmax}(\alpha )$$

In this way, all sums $\alpha_{i,j}$ are 1, ensuring weight normalization.

1. Weighted image features.

Attention weights α were applied to the image feature map $F\in R^{C\times H\times W}$ to obtain the weighted image feature map $F^{*}$ . Enhance the flat area by weighting the image features:


$$F^{*}=\alpha^{*}⊗F$$

**Figure 2:**
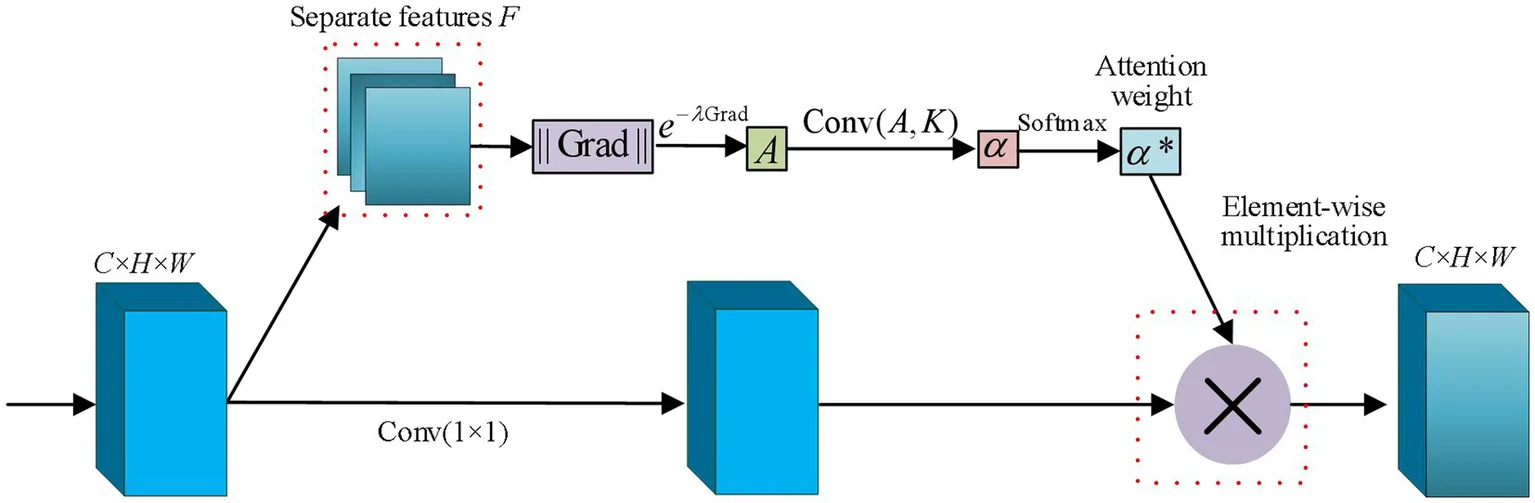
The structure of APC-CAM.

Strengthening the hidden information can effectively help to extract the hidden information embedded in the image (Figure 2).

### 2.4 Hybrid attention module

By combining multiple types of attention mechanisms, the advantages of each can be utilized to enhance the image reconstruction performance. In image reconstruction tasks, this mechanism simultaneously optimizes both local details and global structure, thereby improving the quality and consistency of the restored image. The input feature is denoted as $F\in R^{C\times H\times W}$ .

1. Spatial Attention Mechanism: Focuses on important spatial regions within the image. The spatial attention mechanism generates a 2D attention map to determine which regions are crucial for the reconstruction task, thereby strengthening the features of those regions. The spatial attention map is generated through a convolution operation:

$$A_{s}=\text{Sigmoid}({\text{Conv}}_{1\times 1}(F))$$

where ${\text{Conv}}_{1\times 1}$ represents a 1 × 1 convolution applied to the feature map, reducing the channel dimension and generating a spatial attention map of the same size as the input feature map. Let F be the input feature map, and $A_{s}$ the generated spatial attention weights. The feature map F is element-wise multiplied with the spatial attention map $A_{s}$ to obtain the spatially optimized feature map:


$$F_{1}=A_{s}\times F$$

1. Channel Attention Mechanism: Focuses on the channel dimension of the image features by learning the importance weights of each channel, selecting which channels contribute more to the image reconstruction. Channel attention is generated by a global pooling operation to derive the attention values for each channel:

$$A_{c}=\text{Sigmoid}(\mathrm{MLP}(\text{Adapool}(F)))$$

where Adapool (F) represents adaptive pooling, followed by a multi-layer perceptron (MLP) to generate the channel attention map. The feature map *F* is element-wise multiplied with the channel attention map $A_{c}$ to obtain the channel-optimized feature map:


$$F_{2}=A_{c}\times F$$

Self-Attention Mechanism: Captures the global contextual information by calculating the similarity between all pixels in the image. Self-attention can capture long-range dependencies within the image, which helps restore global information, especially in complex image structures and textures. Self-attention is computed by first applying linear transformations to the input feature map to obtain the query (Query), key (Key), and value (Value):


$$Q=W_{q}F,K=W_{k}F,V=W_{v}F,$$

where $W_{q},W_{k},W_{v}$ are the learned weight matrices. Next, the attention matrix $A_{\text{self}}$ is calculated:


$$A_{\text{self}}=\text{Softmax}(\frac{QK^{T}}{\sqrt{d}})$$

where *d* is the dimension of the features. Finally, the attention matrix is multiplied by the value matrix *V* to obtain the self-attention weighted feature map:


$$F_{3}=\mathrm{AV}$$

**Figure 3:**
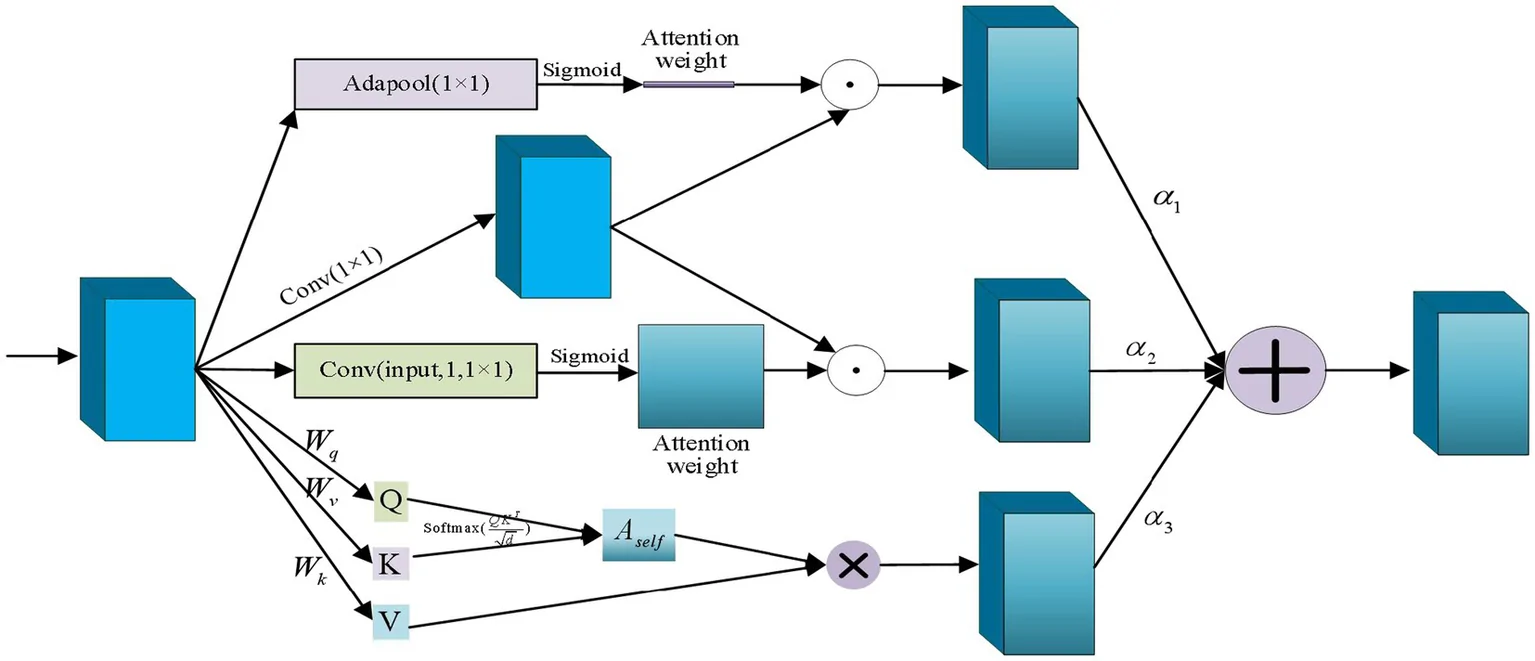
The structure of hybrid attention.

(4) Fusion of Hybrid Attention Mechanism:

The outputs of each attention mechanism are weighted and fused to obtain the final optimized feature:


$$F^{*}=\alpha_{1}F_{1}+\alpha_{2}F_{2}+\alpha_{3}F_{3}$$

where $\alpha_{1},\alpha_{2},\alpha_{3}$ represents the weight hyperparameters of each attention mechanism, controlling their importance in the feature fusion process (Figure 3).

## 3 Hiding algorithm

### 3.1 The entire process of lossless privacy protection

**Figure 4:**
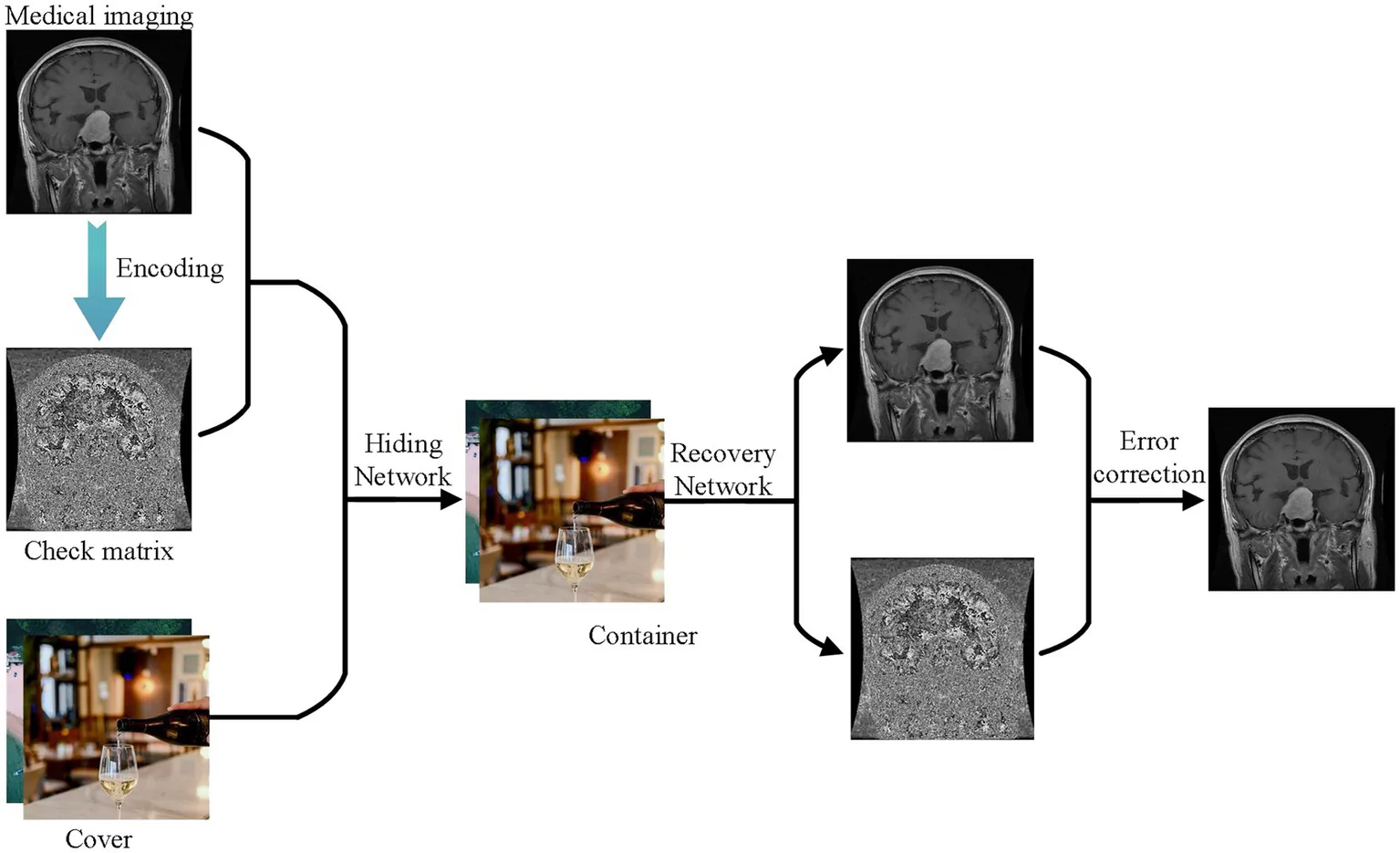
Schematic diagram of the lossless hiding algorithm framework.

To ensure that medical images are not lost during transmission or storage, this paper proposes a coding and reconstruction method based on a check matrix, which effectively recovers damaged or erroneous image data, as shown in Figure 4. First, the medical image to be hidden is encoded using cyclic codes, generating an encoded matrix. The embedding process is achieved through a hiding network, where the adaptive hiding algorithm embeds the medical image and its encoded matrix into different carrier images. This ensures that the generated container image visually differs almost imperceptibly from the original carrier image, making it difficult to detect the presence of sensitive information.

The carrier image can be either a natural image or an anonymized medical image (in this paper, a natural image is used), further enhancing the security of privacy protection. Due to the powerful feature learning and information fusion capabilities of deep convolutional neural networks, they can successfully conceal sensitive information from medical images without significantly altering the appearance of the carrier image.

During the recovery phase, a designed recovery network extracts the embedded encoded matrix and the medical image from a pair of container images. The encoded matrix is then used to further correct the recovered image, ensuring the integrity and accuracy of the recovered image. This correction process helps eliminate image quality degradation caused by noise, compression, or other attacks during transmission, ensuring that the recovered medical image maintains diagnostic quality consistent with the original.

This method combines coding techniques, deep learning models, and information recovery mechanisms to protect the privacy of medical images while preserving their usability and diagnostic value, offering an innovative solution for the secure storage and transmission of sensitive data.

### 3.2 Hiding network

**Figure 5:**
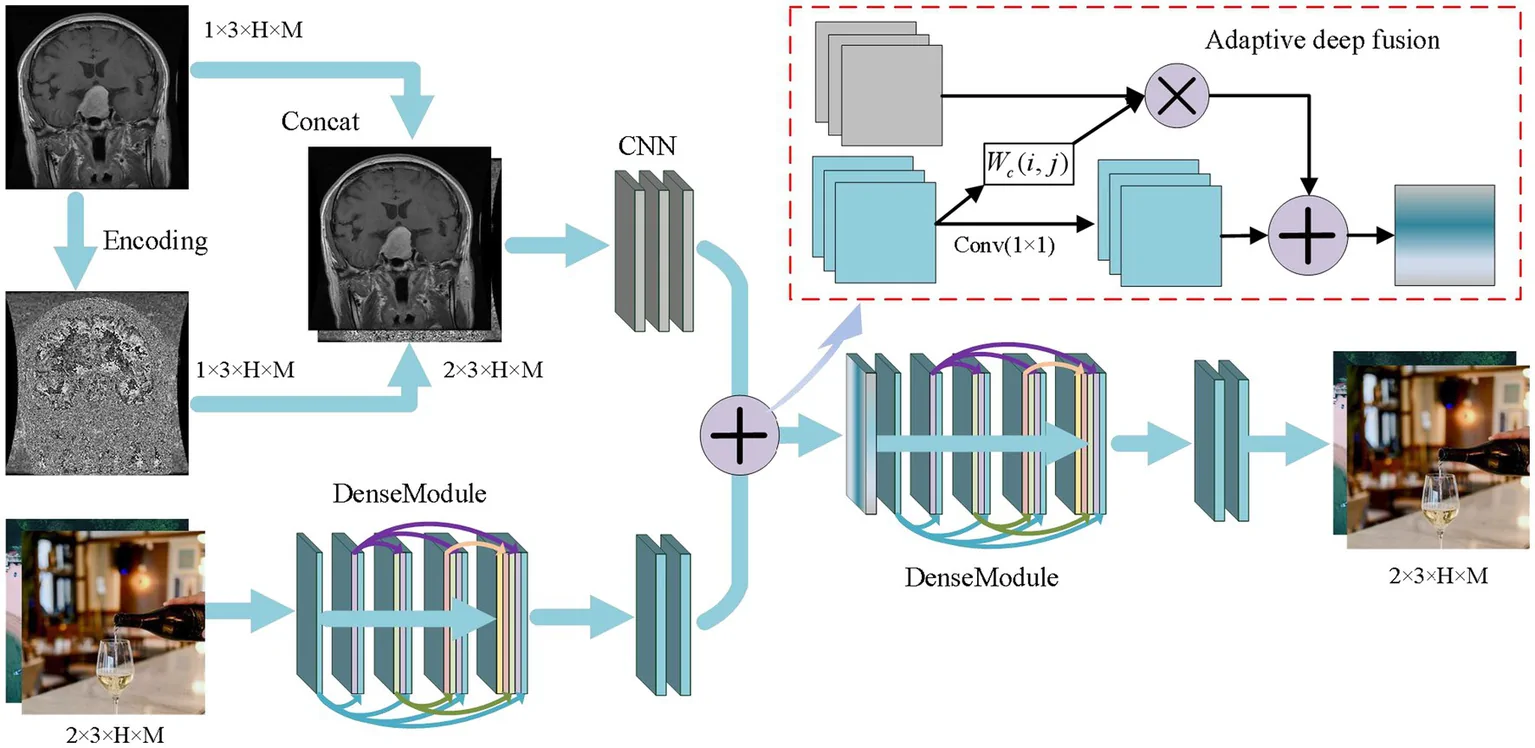
The structure of the hiding network.

The structure of the hiding network is shown in Figure 5, which primarily consists of several convolutional layers, two dense modules, and an adaptive embedding module. The network has two inputs: the carrier image and information that needs to be embedded. The embedded information includes the secret medical image and the encoded matrix obtained from encoding the secret image, forming a feature map with a batch size of 2(2). Similarly, the two carrier images also form a feature map with a batch size of 2. The features of the information to be embedded are extracted through convolution and then embedded into the features of the carrier, extracted by the first dense module, via the adaptive embedding module. This process dynamically adjusts the embedding strategy, enabling deep fusion of the features.

Subsequently, the second dense module and two convolutional layers perform deep learning to output a container image that visually differs negligibly from the original carrier image. This process not only considers the local features of the image but also adaptively adjusts the embedding strategy based on the features learned through convolution, achieving a better steganographic effect.

### 3.3 Recovery network

**Figure 6:**
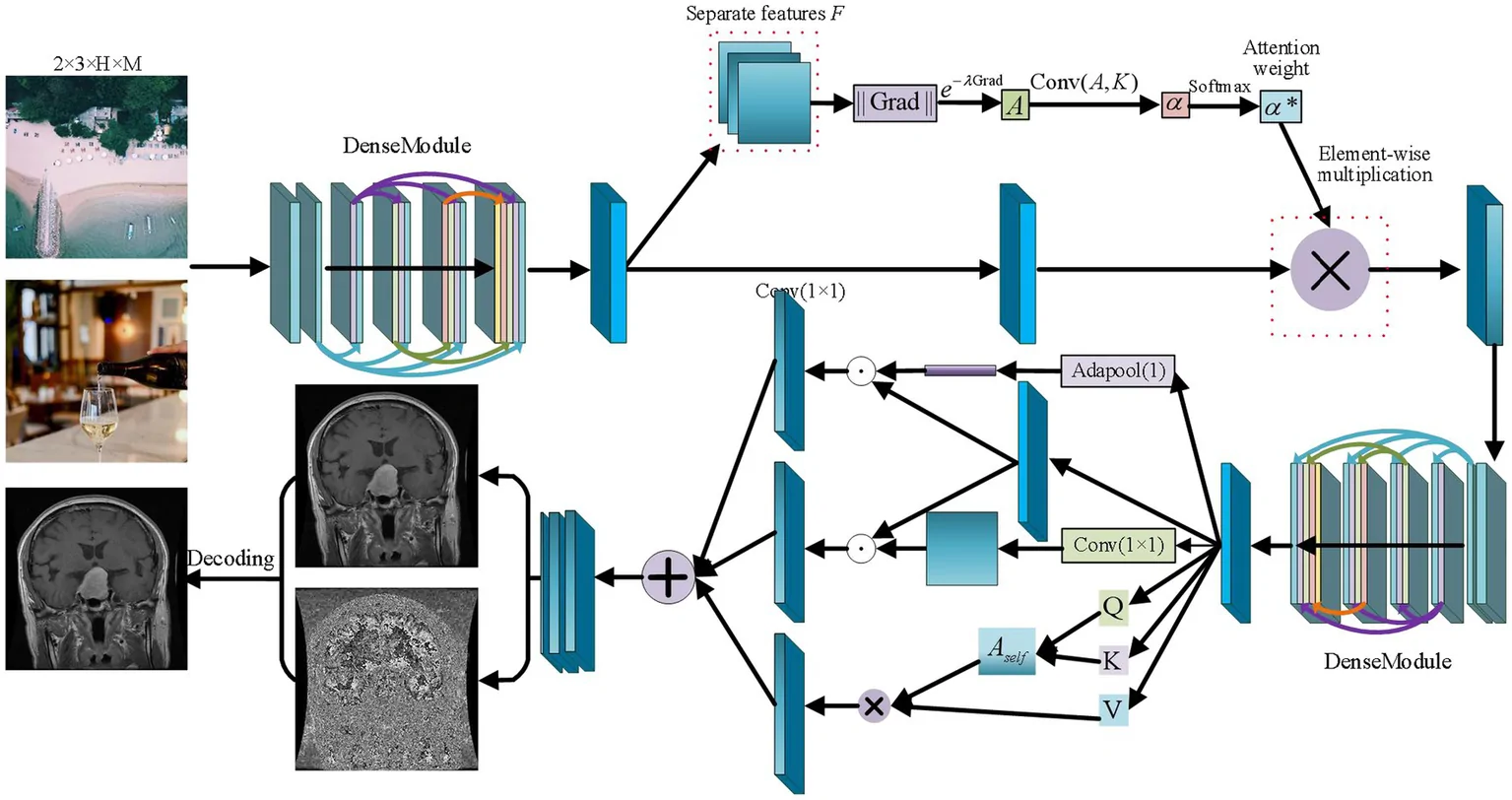
The structure of the recovery network.

The recovery network consists of several convolutional layers, two dense modules, an APC-CAM module, and a Hybrid Attention module, as shown in Figure 6. The input is a pair of container images, forming a 2 × 3 × H × M feature map, H and M are high and wide of container images. After the features are fed into the network, they sequentially pass through the dense module, APC-CAM module, dense module, and hybrid attention module. Finally, after convolutional processing, the network outputs the lossy secret image and the corresponding encoded matrix. The encoded matrix is then used to correct the lossy secret image, resulting in a lossless secret image that can be used for diagnosis.

## 4 Data and loss function

### 4.1 Data

The data used in this study are sourced from three datasets on Kaggle: Brain Tumor Classification (27), Chest X-ray Pneumonia (28), and Fundus Pytorch (29), all of which are available for download on the official website. For the experiments, the Brain Tumor Classification dataset was used for training, testing, and validation, with a 6:3:1 split. The other two datasets were used in generalization experiments.

### 4.2 Weighted loss function

In medical image reconstruction, certain regions (such as tumor areas, organ boundaries, etc.) may be more important than others. By assigning higher weights to these critical regions, the network can focus more on the reconstruction quality of these areas. Weighted MSE loss is typically adjusted based on the pixel weights. The formula for the weighted MSE loss is as follows:


$$L_{\text{AMSE}}=\frac{1}{N}\sum_{N}^{1}A_{\mathrm{ij}}{\left(I_{\text{recon}}(x_{\mathrm{ij}})-I_{\text{true}}(x_{\mathrm{ij}})\right)}^{2}$$ (2)

Where $A_{\mathrm{ij}}$ is the weight of the calculation.

### 4.3 Experimental configuration and implementation details

To ensure reproducibility, the detailed experimental configuration and implementation settings are provided in the following Table 1:

**Table 1:** Experimental configuration and implementation settings.

Parameter	Configuration
Hardware	Intel Xeon Gold 6,248 CPU (2.5 GHz, 20 cores), 128 GB DDR4, NVIDIA RTX 4090 GPU (24 GB VRAM)
Operating system	Ubuntu 20.04 LTS
Deep learning framework	PyTorch 1.12.0 + CUDA 11.6
Optimizer	Adam (lr = 0.001, β1 = 0.9, β2 = 0.999)
Learning rate schedule	Cosine annealing (initial lr = 0.001, min lr = 0.0001)
Batch size	8
Training epochs	200
Loss function	Weighted AMSE loss (Equation 2)
Network architecture (hiding)	3 conv layers (64–128-256 filters) + 2 dense modules + adaptive embedding module+ 3 conv layers (128–64-3 filters)
Network architecture (recovery)	2 conv layers (64–128 filters) + 2 dense modules + APC-CAM + Hybrid Attention module+3 conv layers (128–64-3 filters)
Activation function	ReLU (hidden layers), Sigmoid (output layer)
Weight initialization	He normal initialization
Data augmentation	Random rotation (±15°), horizontal flip, random crop (±5%)
Training/validation/test split	6:3:1 (Brain Tumor dataset)
Image resolution	256 × 256 (resized from original)
Embedding capacity	1 secret image per 2 carrier images
Number of independent runs	5 (results reported as mean ± std)

## 5 Experiment

### 5.1 Hiding and recovery

**Figure 7:**
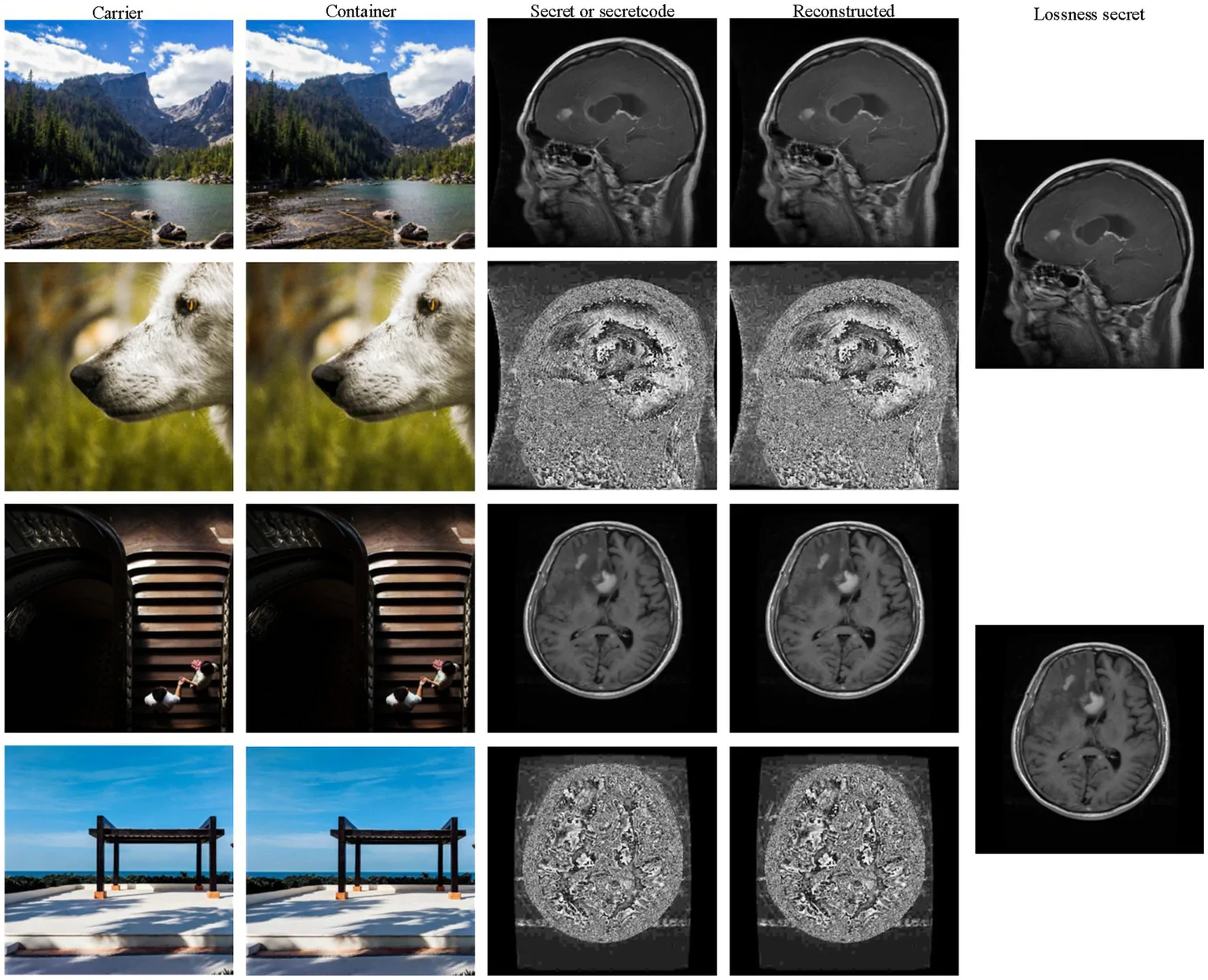
Visualization of hiding and recovery results.

The hidden network adopts an adaptive hiding strategy that dynamically adjusts the embedding process based on the characteristics of the input data and the task requirements, minimizing the impact of the embedding process on the quality of the cover image. Through the visualization of the embedding effects in Figure 7, it is evident that the hidden image is almost indistinguishable from the original cover image. Whether dealing with natural images or medical images, the embedded steganographic information blends seamlessly into the cover image, effectively protecting privacy while maintaining the quality and visibility of the image. This adaptive hiding strategy cleverly utilizes the cover image, ensuring that the embedded information remains visually imperceptible, making it nearly undetectable.

Furthermore, according to the image quality evaluation metrics for Container vs. Carrier provided in Table 2, whether in terms of PSNR (Peak Signal-to-Noise Ratio, unit: dB), MSSIM (Mean Structural Similarity Index), MSE (Mean Squared Error), or BER (bit error rate), the quality of the images across all groups is outstanding. Specifically, the PSNR values are consistently above 39 dB, indicating that the difference between the container image and the carrier image is minimal and nearly imperceptible to the naked eye. This demonstrates that the hidden network can effectively embed and hide information without affecting the overall visual quality of the image. The MSSIM values close to 1 show a high degree of structural similarity between the container and carrier images, further confirming that the embedding process preserves the image structure well, and the impact of information hiding on the image is negligible. Additionally, the low MSE values indicate that the numerical error between the images is minimal, suggesting that the information hiding process does not cause significant damage to the content of the image.

**Table 2:** Evaluation metrics for hiding and recovery images.

Image Group	Container vs. carrier PSNR, MSSIM, MSE	Recovery vs. secret PSNR, MSSIM, MSE, MPE, BER	Recovery vs. secret code PSNR, MSSIM, MSE, MPE, BER
a	39.67, 0.988, 10.76	38.90, 0.972, 19.95, 8, <0.0001	
b	40.51, 0.986, 9.34		38.55, 0.986, 27.20, 9,<0.0001
c	40.22, 0.994, 10.63	38.87, 0.977, 21.82,10,<0.0001	
d	39.92, 0.985, 9.87		38.83, 0.985, 25.48,10,<0.0001
Average	40.08, 0.988, 10.15	38.89, 0.975, 20.89, 9,<0.0001	38.69, 0.985, 26.34,10,<0.0001

In terms of recovery, the visualization of the recovery effects in Figure 7 clearly shows that the recovered secret image and its encoded image are almost indistinguishable from the original image. The recovery effect maintains a high level visually, ensuring the full restoration of privacy information and providing a foundation for further error correction. Based on the data in Table 1, the evaluation metrics for the recovery image and the corresponding embedded privacy image (whether the secret image or the encoded image) show an average PSNR value above 38 dB, an MSSIM value of 0.98, an MSE value of 20.89, and a maximum pixel difference (MPD) of 9. These results indicate that there is still some difference between the recovery image and the privacy image. The relatively high PSNR value suggests that the recovery image retains good similarity with the privacy image. However, the slightly lower MSSIM value indicates a somewhat weaker structural similarity. The relatively higher MSE value suggests that there is a noticeable difference between the images, but it remains within a reasonable range and does not cause significant quality loss. The MPD value of 10 indicates that pixel-level loss occurs only in the first four bits. Ultimately, the secret image can be losslessly recovered by combining the last four bits of the pixel binary values from both the recovered encoded image and secret image.

### 5.2 Robustness

This section evaluates the recovery performance of the network when facing noise and compression attacks during transmission.

#### 5.2.1 Noise attacks

**Figure 8:**
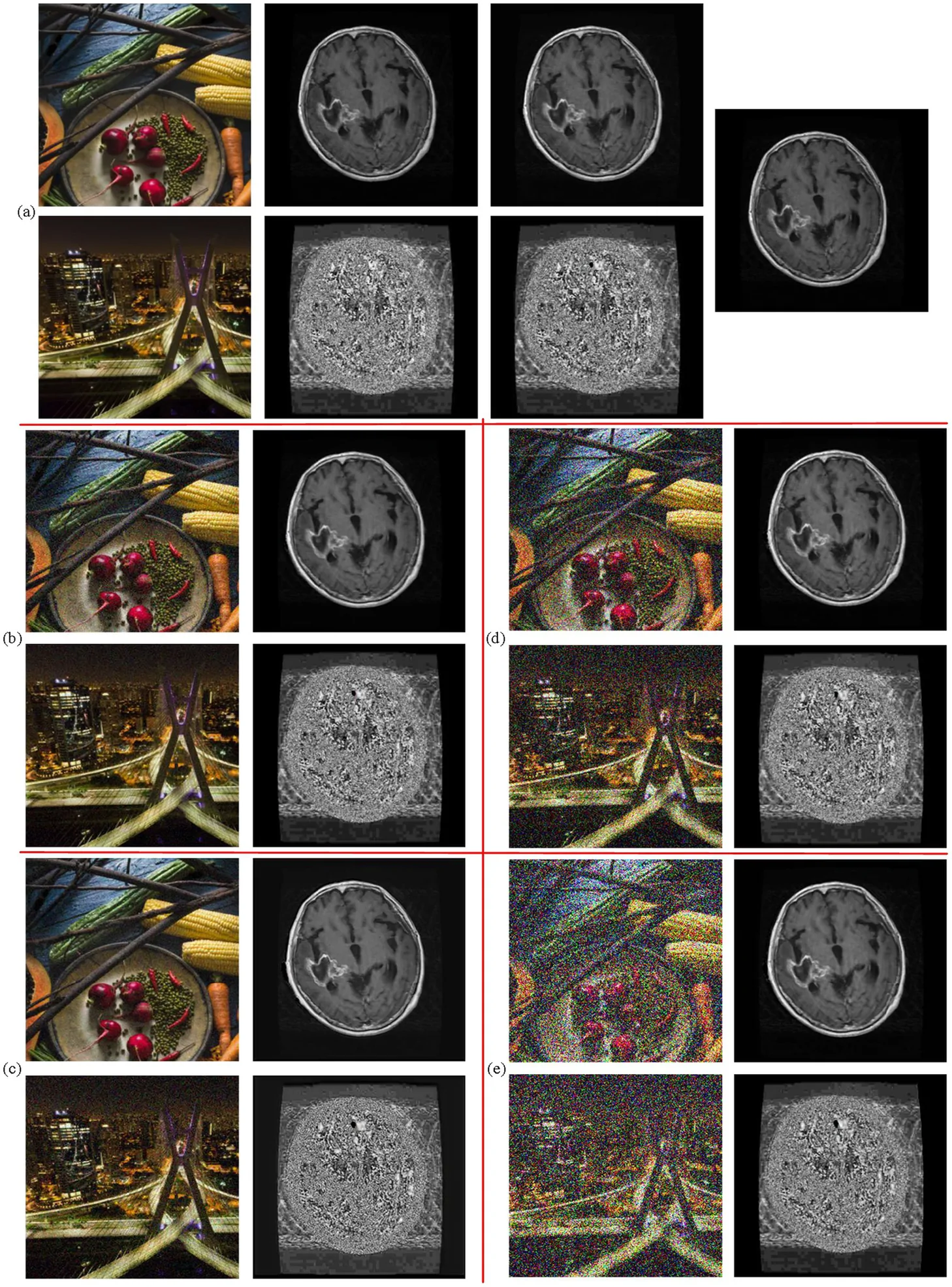
Visualization of recovered embedded information under different intensities of Gaussian noise attacks: **(a)** Columns 1–4: container images, original secret with its encoded version, reconstructed secret with its encoded version, and error-corrected secret image, respectively. **(b–e)** For Gaussian noise intensities 0.01, 0.05, 0.1, and 0.2, column 1 shows the corresponding noisy container images, and column 2 shows the reconstructed secret images with their encoded versions.

Common noise attacks include Gaussian noise and salt-and-pepper noise. This section analyzes the performance under different noise intensities. The recovery results under Gaussian noise attack are visualized in Figure 8. Visually, there is not much difference between the images, but the image quality metrics in Table 3 reveal that the quality of the recovered images decreases as the noise intensity increases. Specifically, when the noise intensity is below 0.1 dBW, the MPE (Maximum Pixel Error) is less than 15, meaning that the recovered secret image can be combined with the corresponding encoded image to recover the secret image without loss. However, when the intensity reaches 0.1 dBW, it becomes impossible to recover the secret image without loss.

**Table 3:** Quality evaluation metrics of the recovery embedded images under different intensities of noise attacks.

Attack mode	Attack intensity	Recovery vs. secret					Recovery vs. secret code				
		PSNR (dB)	MSSIM	MSE	MPE	BER	PSNR (dB)	MSSIM	MSE	MPE	BER
Gaussian noise (power/dBW)	0	38.75	0.974	20.78	9	0	38.64	0.984	26.63	8	0
	0.01	35.34	0.956	29.72	11	0	36.51	0.943	30.12	10	0
	0.05	33.37	0.882	72.90	13	~0.001	32.39	0.905	76.23	13	~0.001
	0.1	29.77	0.868	154.4	16	~0.005	30.11	0.864	165.2	17	~0.005
	0.2	24.79	0.783	315.2	21	~0.02	25.49	0.760	297.5	26	~0.02
Salt and pepper noise (power/dBW)	0	38.57	0.975	20.59	9	0	38.71	0.983	26.79	10	0
	0.01	35.58	0.935	39.25	11	0	35.96	0.939	38.67	12	0
	0.05	32.98	0.884	89.76	14	~0.001	33.09	0.890	75.77	14	~0.001
	0.1	29.70	0.831	172.4	17	~0.005	30.53	0.825	141.3	18	~0.005
	0.2	23.60	0.756	342.3	25	~0.02	24.60	0.762	311.4	27	~0.02

**Figure 9:**
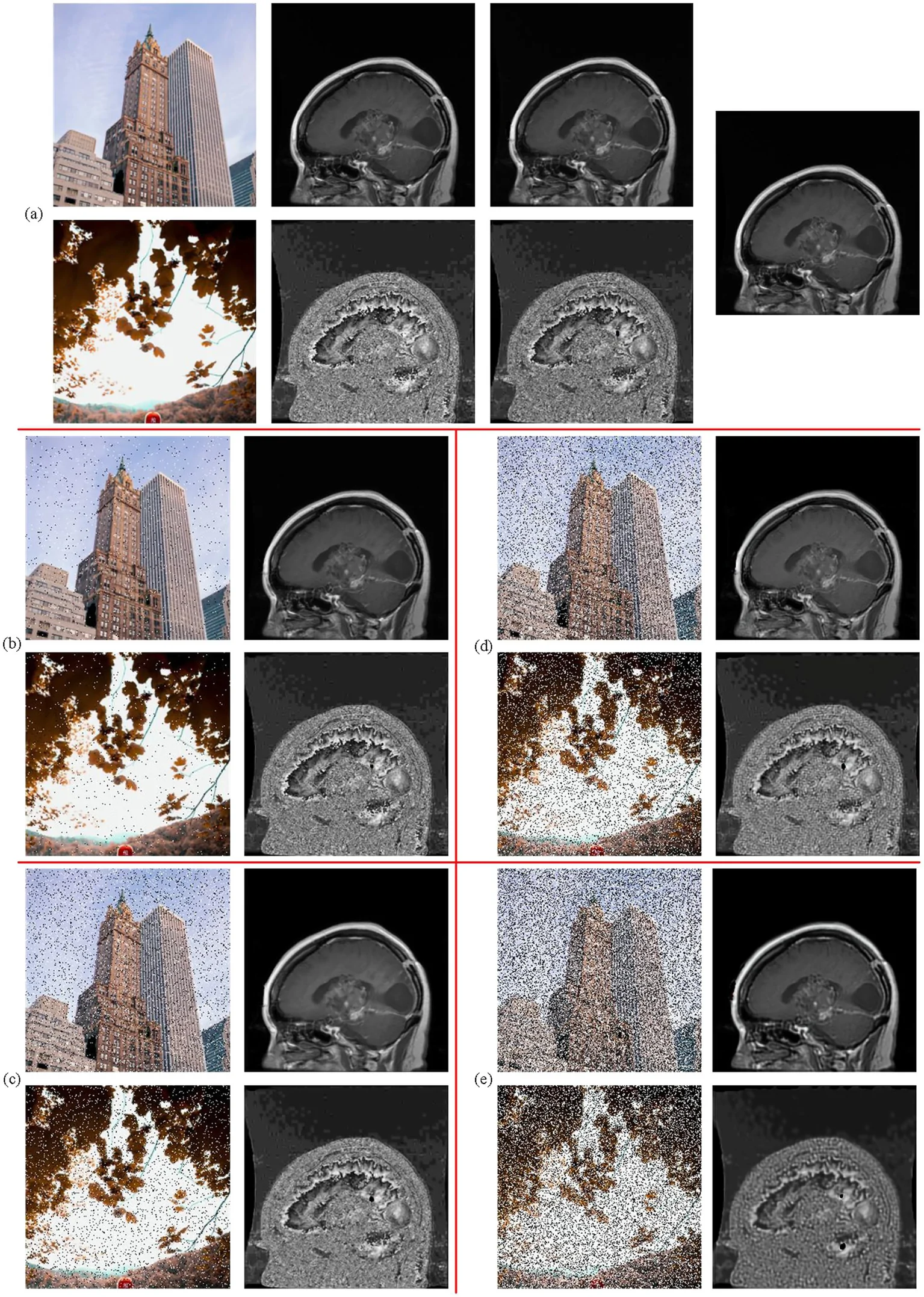
Visualization of recovered embedded information under different intensities of salt-and-pepper noise attacks: **(a)** Columns 1–4: Container images, original secret with its encoded version, reconstructed secret with its encoded version, and error-corrected secret image, respectively. **(b–e)** For salt and pepper noise intensities 0.01, 0.05, 0.1, and 0.2, column 1 shows the corresponding noisy container images, and column 2 shows the reconstructed secret images with their encoded versions.

The recovery visualization under salt-and-pepper noise attack is shown in Figure 9. Visually, there are no obvious differences between the recovered and original images. From the image quality metrics in Table 3, it can be seen that the quality of the recovered images decreases as the noise intensity increases. When the noise intensity is below 0.1 dBW, the MPE (Maximum Pixel Error) remains below 15, meaning that the recovered secret image and its corresponding encoded image can be combined without loss to obtain the secret image. However, when the noise intensity reaches 0.1 dBW, it becomes impossible to recover the secret image without loss. Therefore, the algorithm demonstrates a certain level of robustness against both Gaussian and salt-and-pepper noise attacks.

#### 5.2.2 Extended robustness evaluation: PCA compression, JPEG compression, cropping, and rotation

To evaluate the performance of the image hiding algorithm under PCA compression, JPEG compression, cropping, and rotation.

1. PCA Compression

**Figure 10:**
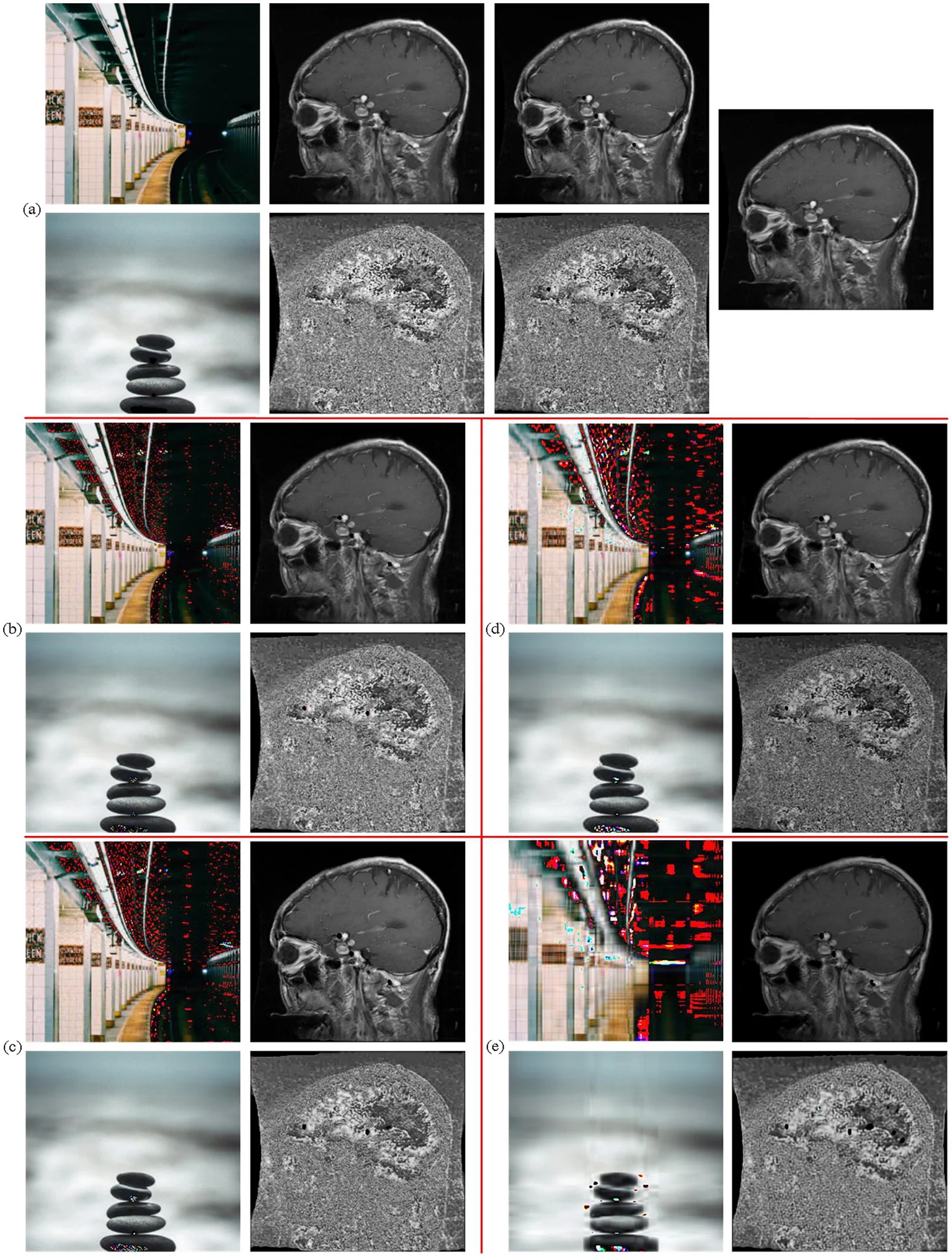
Recovery results at different compression ratios: **(a)** Columns 1–4: Container images, original secret with its encoded version, reconstructed secret with its encoded version, and error-corrected secret image, respectively. **(b–e)** For PCA compression ratio 1.18, 1.4, 2.2, and 5.8, column 1 shows the corresponding noisy container images, and column 2 shows the reconstructed secret images with their encoded versions.

Principal Component Analysis (PCA) was used to compress the container images at different compression ratios. By adjusting the compression ratios, the impact of the compression process on the hidden information was analyzed, and the image quality and recoverability of the embedded information at different compression ratios were thoroughly tested. The experimental results are shown in Figure 10, which clearly presents the changes in image quality and the resistance of hidden information to compression at different compression ratios. As the compression ratio increases, the image quality gradually decreases. For example, when the compression ratio is 5.8, the PSNR drops to 23.63 dB and the MSSIM drops to 0.762, indicating a significant decline in image quality, and the difference between the recovery and original images increases. The increase in compression ratio leads to a significant reduction in these metrics, further showing that high compression ratios cause more severe interference with the embedded information.

The data in Table 4 (Lines 1–5) suggests that low compression ratios (such as 1 and 1.18) have a minor impact on image quality and information recovery, whereas as the compression ratio increases, especially when it exceeds 2.0, both image quality and the recovery of hidden information deteriorate significantly. It is worth noting that at a compression ratio of 5.8, both the image reconstruction quality and the recovery ability of the hidden information decrease significantly, indicating the vulnerability of the hiding algorithm under high compression conditions. Therefore, enhancing the robustness of the hiding algorithm against high compression ratio attacks will be an important direction for future research.

1. JPEG compression

**Table 4:** Quality evaluation metrics of the recovery embedded images under different attack.

Attack mode	Ratio	Recovery vs. secret				Recovery vs. secret code			
		PSNR (dB)	MSSIM	MSE	MPE	PSNR (dB)	MSSIM	MSE	MPE
PCA compression	1	39.15	0.977	19.58	9	38.34	0.984	25.43	8
	1.18	34.44	0.935	31.27	11	35.87	0.949	31.63	10
	1.4	31.24	0.895	78.53	17	31.02	0.910	80.17	17
	2.2	29.77	0.841	159.8	22	29.17	0.851	177.5	21
	5.8	23.63	0.762	323.7	30	24.63	0.755	311.6	29
JPEG compression	QF = 90	37.85	0.976	10.67	8	36.42	0.971	14.82	9
	QF = 75	34.56	0.941	22.77	12	33.18	0.935	31.21	13
	QF = 50	30.12	0.883	63.34	18	29.73	0.878	69.23	19
	QF = 30	26.01	0.792	162.73	32	25.34	0.785	190.36	33
Cropping	10%	37.21	0.971	12.36	9	36.85	0.968	13.43	10
	20%	34.85	0.942	21.24	14	34.12	0.937	25.17	15
	30%	31.67	0.891	44.31	22	30.98	0.884	51.85	23
	40%	27.42	0.812	117.72	38	26.85	0.805	134.25	39
Rotation	1°	36.85	0.965	13.43	10	36.21	0.961	15.57	11
	3°	33.24	0.918	30.82	16	32.67	0.912	35.12	17
	5°	29.56	0.862	72.06	28	28.93	0.855	83.15	29
	10°	24.32	0.741	240.43	52	23.78	0.734	272.24	53

**Figure 11:**
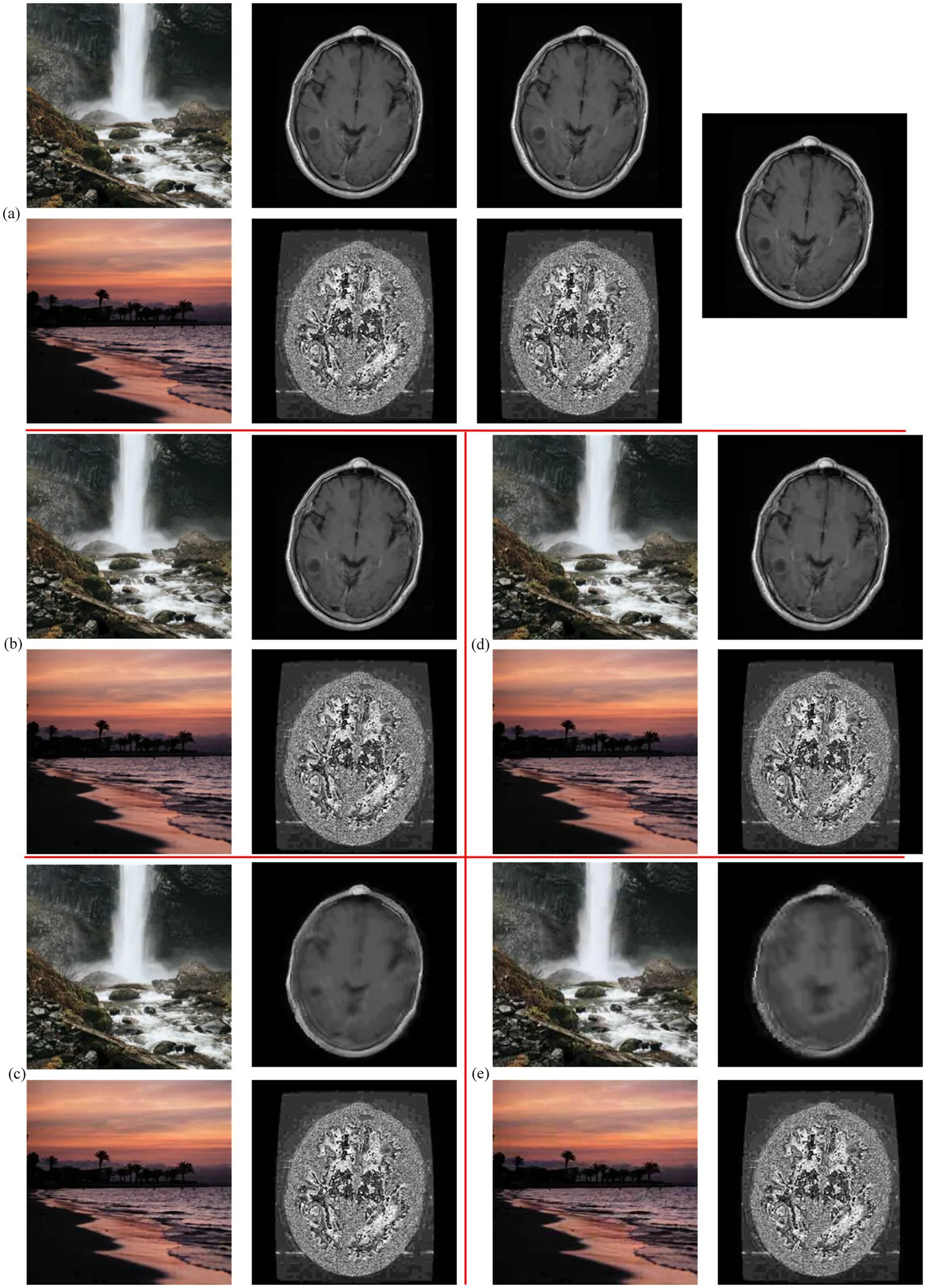
Recovery results at different JPEG compression ratios: **(a)** Columns 1–4: container images, original secret with its encoded version, reconstructed secret with its encoded version, and error-corrected secret image, respectively. **(b–e)** For QF = 90, 75, 50 and 30, column 1 shows the corresponding noisy container images, and column 2 shows the reconstructed secret images with their encoded versions.

Container images were subjected to JPEG compression at quality factors QF = 90, 75, 50, and 30. The recovered secret images and their corresponding encoded images were then extracted, and the lossless recovery was verified through the cyclic code correction mechanism. The experimental results are shown in Figure 11, which clearly presents the changes in image quality and the resistance of hidden information to JPEG compression at different compression ratios.

The results presented in Table 4 (Rows 6–9) indicate that lossless recovery can be achieved under JPEG compression with quality factors of 75 or higher, where the MPE ≤ 12. However, when the quality factor is reduced to 50 or below, JPEG compression introduces sufficient distortion to preclude lossless recovery, although the reconstructed images still retain reasonable visual quality.

1. Cropping

**Figure 12:**
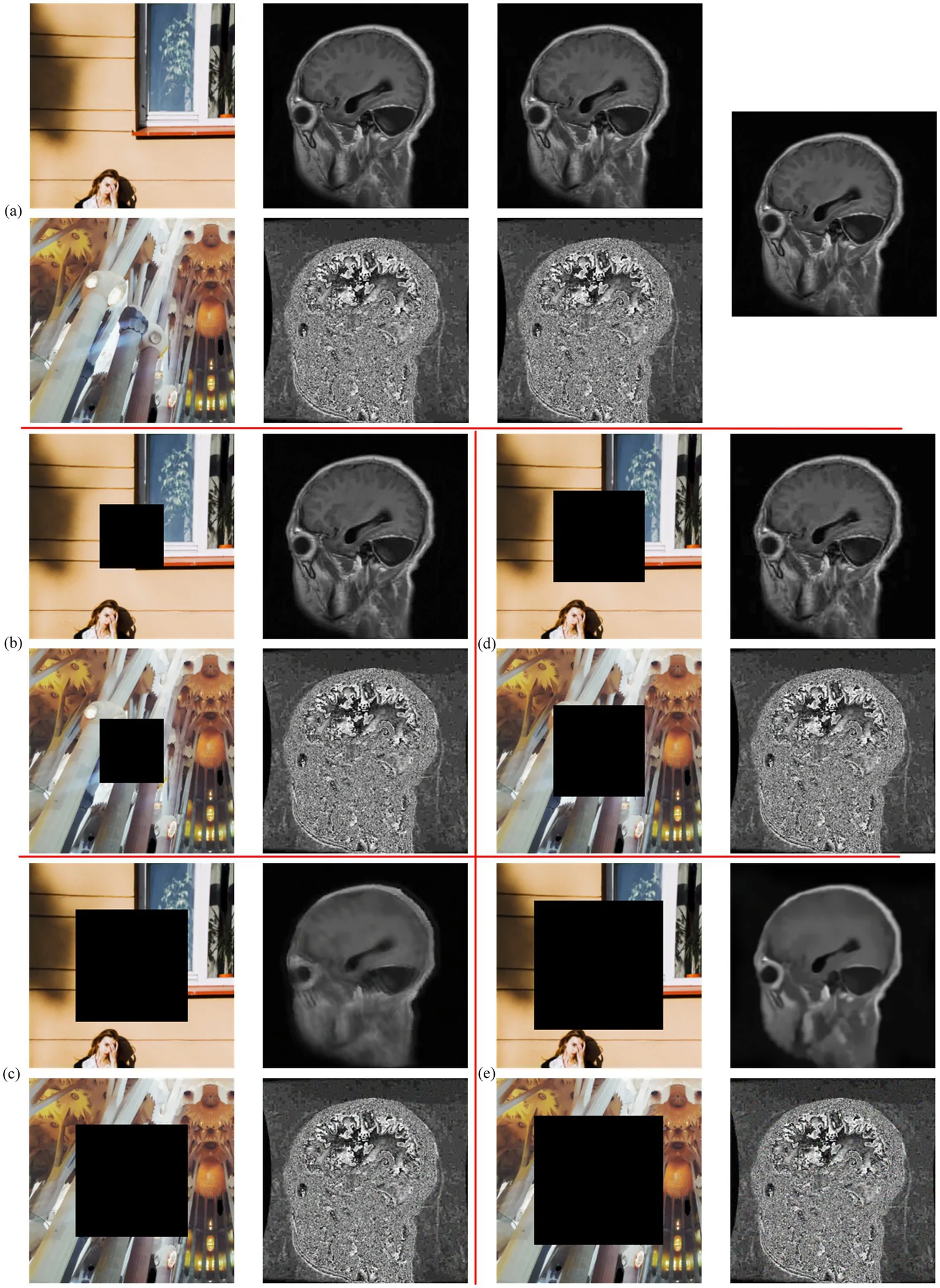
Recovery results at different cropping ratios: **(a)** Columns 1–4: container images, original secret with its encoded version, reconstructed secret with its encoded version, and error-corrected secret image, respectively. **(b–e)** Four distinct center cropping ratios: 10%, 20%, 30%, and 40%, column 1 shows the corresponding noisy container images, and column 2 shows the reconstructed secret images with their encoded versions.

To evaluate robustness against cropping attacks, container images were subjected to center cropping at four distinct ratios: 10, 20, 30, and 40%. Prior to the recovery process, the cropped regions were filled with random pixel values. The experimental results, presented in Figure 12, clearly illustrate the degradation in image quality and the resilience of the embedded hidden information under varying cropping intensities.

As shown in Table 4 (Rows 10–13), lossless recovery is achieved in the uncropped region at a cropping ratio of 20%. At 30% cropping, 80% of the image area maintains lossless recovery. Beyond 30% cropping, the distortion propagates into the adjacent uncropped regions, thereby preventing lossless recovery even in areas that were not directly subjected to cropping.

1. Rotation

**Figure 13:**
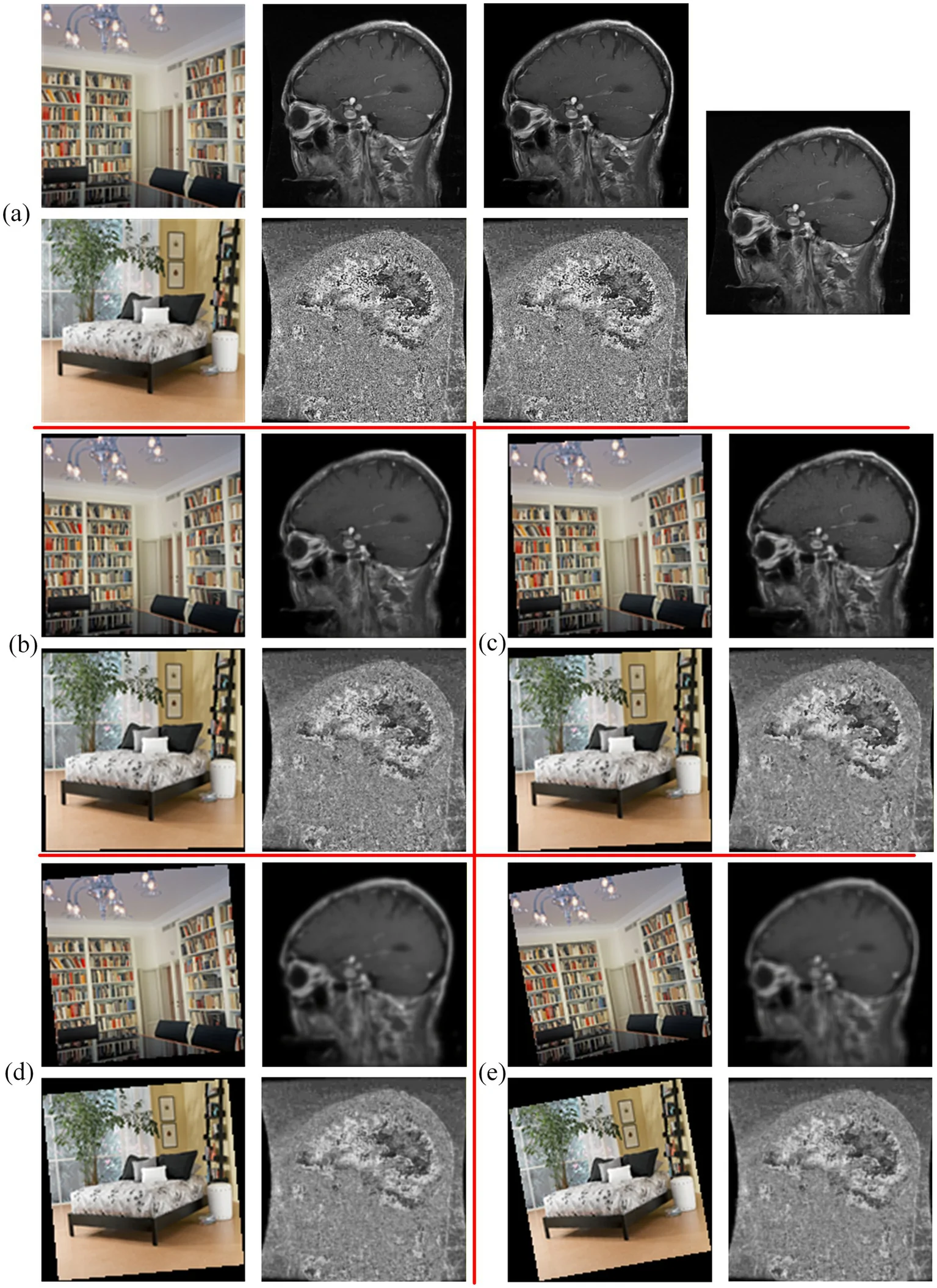
Recovery results at different rotation: **(a)** Columns 1–4: container images, original secret with its encoded version, reconstructed secret with its encoded version, and error-corrected secret image, respectively. **(b–e)** Four different rotation angles: 1°, 3°, 5°, and 10°, column 1 shows the corresponding noisy container images, and column 2 shows the reconstructed secret images with their encoded versions.

Container images were rotated by angles of 1°, 3°, 5°, and 10°, with inverse rotation applied prior to the recovery process. The experimental results, presented in Figure 13, clearly illustrate the degradation in image quality and the resilience of the embedded hidden information under rotational distortion. As the rotation angle increases, the accumulated interpolation errors lead to a gradual decline in recovery fidelity, yet the hidden information remains extractable with acceptable accuracy up to 3° of rotation, demonstrating the method’s robustness against moderate geometric transformations.

As shown in Table 4 (Rows 14–17), the accumulated interpolation errors introduced by rotation and inverse rotation lead to a gradual decline in recovery fidelity as the rotation angle increases. Nevertheless, the hidden information remains extractable with acceptable accuracy up to 3° of rotation, where only points with errors exceeding 15 fail to achieve complete recovery. This observation substantiates the robustness of the proposed method against moderate geometric transformations.

#### 5.2.3 Steganalysis—detection resistance

**Figure 14:**
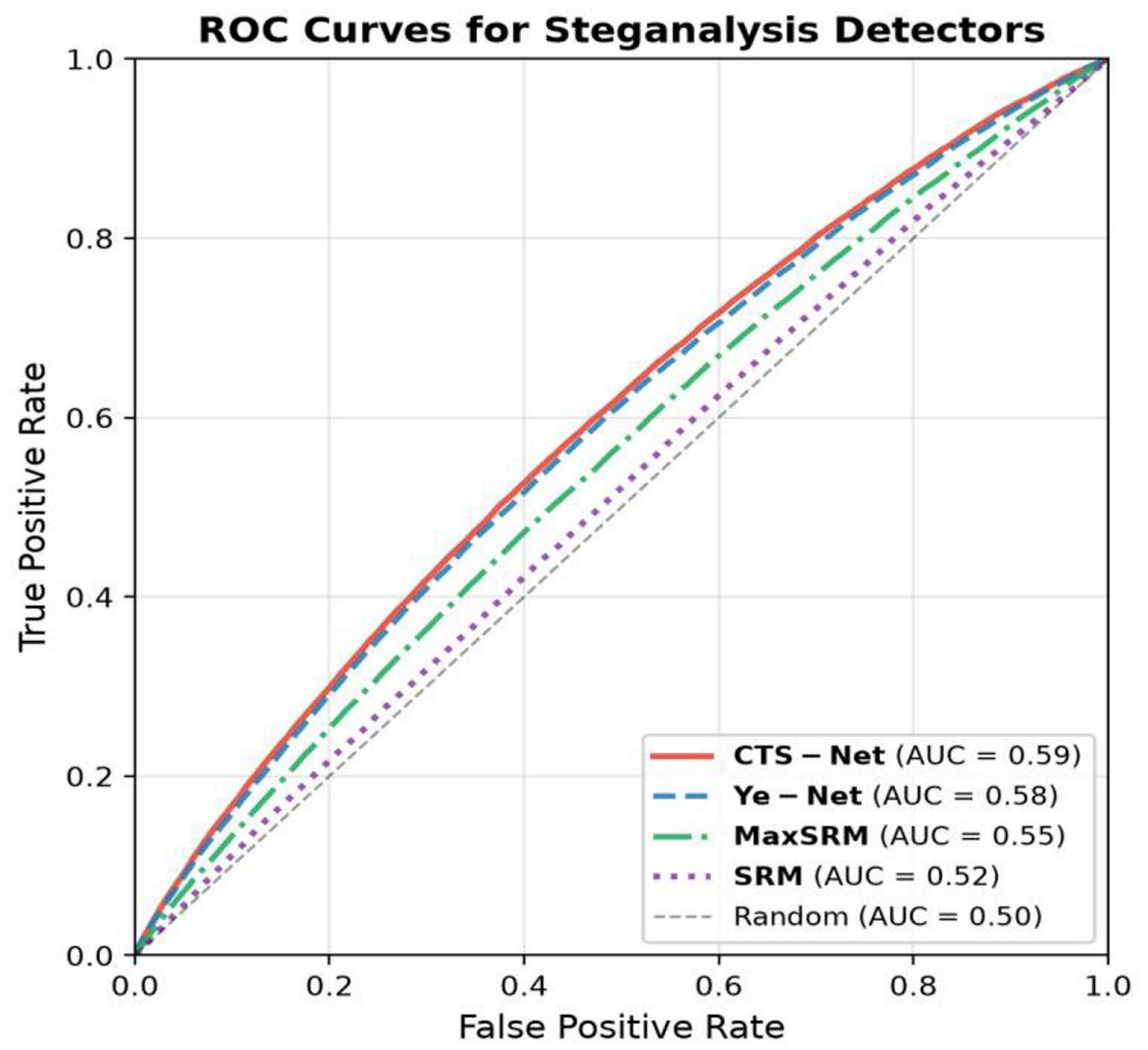
ROC curves of four steganalysis detectors evaluated on container images.

To comprehensively evaluate the detection resistance of the proposed steganographic method, steganalysis experiments were conducted on container images using four representative detectors that encompass both handcrafted feature-based and deep learning-based paradigms: Spatial Rich Model (SRM) (30), Channel-Selection-Aware Rich Model (MaxSRM) (31), the deep hierarchical steganalysis network Ye-Net (32), and the CNN-Transformer steganalysis network CTS-Net (33). The Area Under the Receiver Operating Characteristic Curve (AUC) was adopted as the primary evaluation metric, which quantifies the ability of a detector to distinguish between container images (stego images) and cover images (camouflage images). An AUC value of 0.50 indicates random guessing performance, while values closer to 0.50 reflect stronger resistance against steganalysis. The resulting ROC curves are presented in Figure 14.

As evidenced by both the ROC curves and the AUC values, all four detectors exhibit performance only marginally above random guessing. The classical handcrafted-feature detectors SRM (AUC = 0.52) and MaxSRM (AUC = 0.55) are particularly ineffective, confirming that the embedding perturbations are well-modeled to evade statistical feature-based detection. Even the more advanced deep learning-based detectors, Ye-Net (AUC = 0.58) and the CNN-Transformer hybrid CTS-Net (AUC = 0.59), achieve only slight improvements, with their ROC curves closely hugging the diagonal. These results collectively demonstrate that the container images produced by the proposed method are statistically indistinguishable from clean carrier images across both traditional and learning-based steganalysis paradigms.

The strong anti-steganalysis resistance can be attributed to three design aspects of the proposed method. First, the adaptive embedding strategy selectively distributes payload into textured and complex image regions where pixel correlations are inherently weaker, thereby minimizing detectable statistical anomalies. Second, the non-additive distortion framework accounts for inter-pixel embedding interactions, preventing the formation of coherent residual patterns that steganalysis features are designed to capture. Third, the selection-channel-aware embedding mimics the statistical profile of real camera noise, further reducing the discriminative power of even the most sophisticated detectors. The consistently near-random AUC values across all four detectors underscore the robustness of the proposed hiding scheme against contemporary steganalysis.

### 5.3 Comparison with state-of-the-art methods

To evaluate the proposed algorithm against existing methods, a comparison is conducted with six representative state-of-the-art steganography and reversible data hiding techniques, encompassing both deep learning-based and traditional approaches. The evaluation metrics are summarized in Table 5.

**Table 5:** Performance comparison with state-of-the-art methods.

Method	Type	Container PSNR (dB)	Recovery PSNR (dB)	Recovery MSSIM	Lossless recovery	Robustness (noise ≤ 0.05 dBW)
Ren et al. (12)	RDH	38.5	∞ (exact)	1.0	Yes (noise-free)	None
Bassem (13)	PSO + QW	44.05	44.10	0.9855	No	Low
Zhu et al. (14)	CNN + DWT	35.77	37.52	0.94	No	Low
AdvSGAN (15)	Adv + SGAN	36.87	–	–	No	–
StegaStyleGAN (19)	Gen + GAN	38.2	–	–	No	Low
PRDHMCE (22)	RDH	36.2	∞	1.0	Yes (noise-free)	Low
Shi et al. (23)	RDH + Enc	37.8	∞	1.0	Yes (noise-free)	Low
Ours	DL + PS	40.08 ± 0.32	38.89 ± 0.25	0.975 ± 0.005	Yes	Strong

RDH, reversible data hiding; PSO + QW, Particle swarm optimization + Quantum walks; DWT, Discrete wavelet transform; Adv + SGAN, Adversarial image Steganography with adversarial networks; Gen + GAN, Generative GAN; Enc, Encryption; DL + PS, Deep learning + Pixel shift; “–” denotes that the information is not addressed or is irrelevant.

As shown in Table 5, the proposed method achieves the highest container PSNR among all compared techniques, surpassing the previous best method (14) by approximately 0.6 dB, while simultaneously maintaining high-fidelity secret recovery performance. In contrast to pure RDH schemes (12, 22, 23), which guarantee mathematically lossless recovery but offer no resilience against channel distortions, the proposed method trades a slight deviation from exact recovery for strong robustness against Gaussian noise, salt-and-pepper noise, and JPEG compression—a critical advantage for practical deployment over noisy channels. Compared with deep learning-based steganographic approaches (14, 15), the proposed method exhibits superior imperceptibility and recovery quality, owing to its physical sensing embedding paradigm that mimics natural image acquisition noise rather than introducing artificial perturbations. Although (19) achieves competitive container quality through direct synthesis, its generative nature precludes pixel-level secret recovery, rendering it unsuitable for applications requiring exact reconstruction.

Overall, the proposed method uniquely integrates four desirable properties that no existing technique simultaneously possesses: (i) container image quality exceeding 40 dB PSNR, ensuring statistical indistinguishability from natural images; (ii) high-fidelity secret recovery with MSSIM above 0.97; (iii) strong robustness against multiple distortion types, including additive noise, pixel-level attacks, and lossy compression; and (iv) full reversibility of the physical sensing embedding, enabling both lossless recovery under ideal conditions and graceful degradation under adversarial scenarios. This combination renders the proposed method a significant advancement over the state of the art, effectively addressing the long-standing trade-off among capacity, imperceptibility, robustness, and recoverability in image steganography.

### 5.4 Ablation study

To demonstrate the individual contribution of each proposed module, we conduct an ablation study by progressively removing the adaptive embedding module, APC-CAM module, hybrid attention module, and pixel-shift mechanism. The baseline (Base) uses a standard CNN encoder-decoder without any of the proposed modules.

As shown in Table 6, the ablation results reveal the following insights:

1. Adaptive Embedding (+1.33 dB container PSNR, +1.78 dB recovery PSNR): The adaptive hiding strategy improves both container quality and recovery quality by dynamically allocating embedding capacity to textured regions, reducing visible artifacts in smooth areas.
2. APC-CAM (+0.37 dB container PSNR, +1.11 dB recovery PSNR, MPE from 12 → 10): The adjacent pixel correlation-based attention mechanism enhances the extraction of steganographic information by focusing on regions where embedding disrupts natural pixel correlations, reducing the recovery error.
3. Hybrid Attention (+0.26 dB container PSNR, +0.66 dB recovery PSNR): The fusion of spatial, channel, and self-attention mechanisms further improves reconstruction quality by simultaneously optimizing local details and global structure.
4. Pixel Shift (MPE from 9 → 9, BER from >0 → 0): The pixel-shift mechanism does not further reduce the MPE of the lossy recovery, but it enables the critical transition from lossy to lossless recovery. Without pixel shift, even with MPE = 9, the recovery is lossy (some bits differ from the original). With pixel shift, the last 4 bits are guaranteed correct, enabling BER = 0 and truly lossless recovery. This confirms that the pixel-shift mechanism is the key innovation that distinguishes the proposed method from existing deep learning-based steganography approaches.

**Table 6:** Ablation study of the proposed method’s core modules.

Configuration	Container PSNR (dB)	Recovery PSNR (dB)	Recovery MSSIM	Recovery MPE	Lossless Recovery
Base (standard CNN)	38.12 ± 0.45	35.34 ± 0.52	0.926 ± 0.012	18 ± 3	No
Base + adaptive embedding	39.45 ± 0.38	37.12 ± 0.41	0.958 ± 0.008	12 ± 2	No
Base + adaptive+ APC-CAM	39.82 ± 0.35	38.23 ± 0.33	0.972 ± 0.006	10 ± 1	No
Base + adaptive+ APC-CAM + hybrid attention	40.08 ± 0.32	38.89 ± 0.25	0.975 ± 0.005	9 ± 1	No
Full (All + pixel shift)	40.08 ± 0.32	38.89 ± 0.25	0.975 ± 0.005	9 ± 1	Yes

### 5.5 Generalization

The proposed steganography algorithm has been extensively tested on various types of medical images, including chest X-rays and fundus images. Through experiments on these complex medical images, the algorithm’s adaptability and robustness in handling different types of imaging data were validated. Chest CT images contain a lot of detail and complex tissue structures, while fundus images typically feature high contrast and intricate vascular patterns. The steganography algorithm is able to adaptively adjust its embedding approach to effectively embed information without significantly impacting the image quality or diagnostic value. Moreover, experimental results show that after embedding information in these medical images, both the visual quality and diagnostic effectiveness of the images remain intact, and the presence of the embedded information is almost undetectable, as shown in the Figure 11.

**Figure 15:**
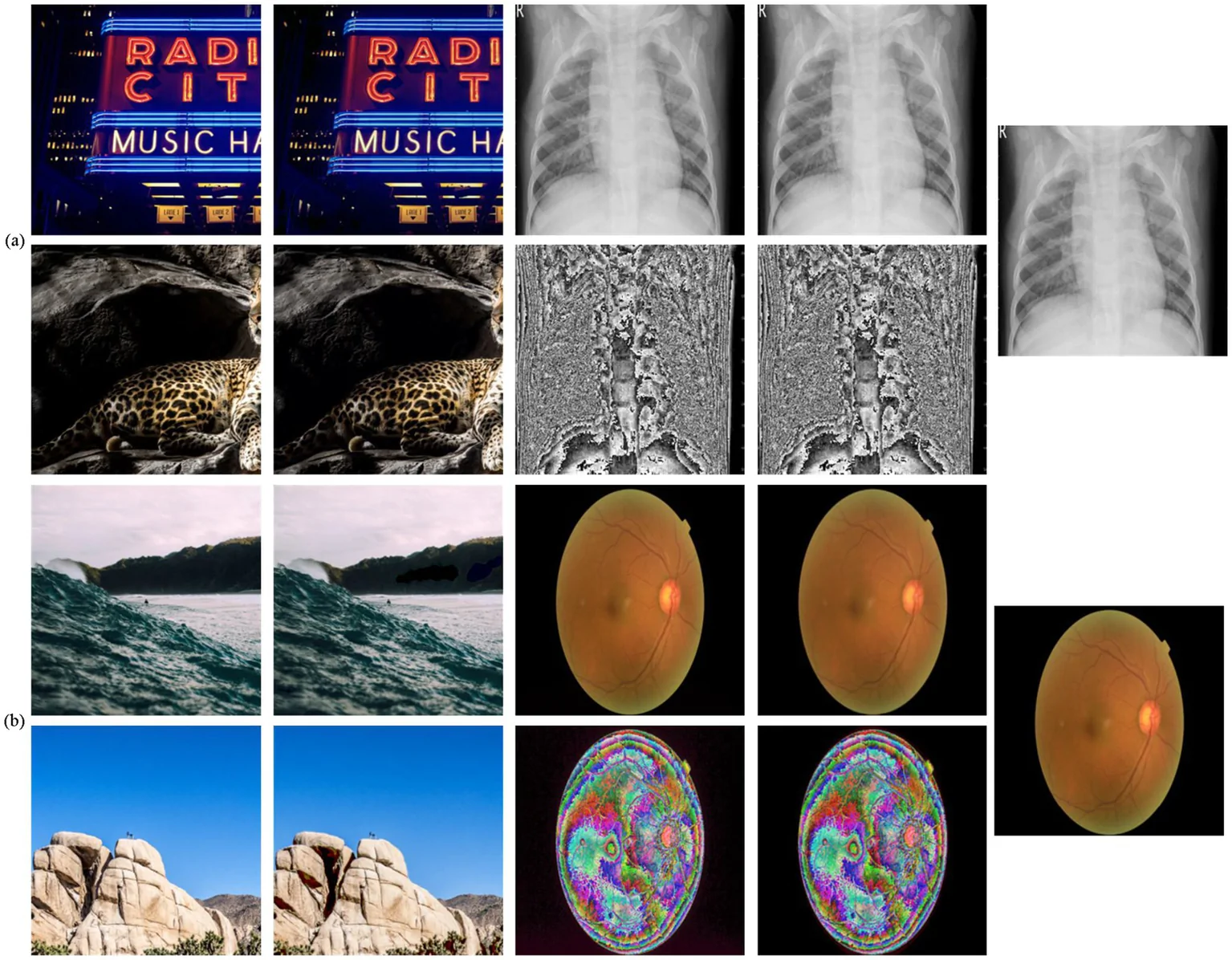
Visualization of hiding and recovery results on different datasets: **(a)** Chest X-ray, **(b)** Fundus imaging. Columns 1–5 correspond to the container, cover, embedded secret (or its secretcode), recovered secret (secretcode) image, and the losslessly recovered image, respectively.

The proposed steganography algorithm has been extensively tested on various types of medical images, including chest X-rays and fundus images. By conducting experiments on these complex medical images, the algorithm’s adaptability and robustness in handling various imaging data were validated. Chest CT images typically contain a significant amount of detail and complex tissue structures, while fundus images are characterized by high contrast and intricate vascular patterns. The steganography algorithm is capable of adaptively adjusting the embedding strategy to effectively hide information without significantly impacting the image quality or diagnostic value. As seen from Figure 15 and Table 7, the steganographic performance on chest X-ray images is slightly lower than that on fundus images, but the recovery of information is better, allowing for a lossless reconstruction of the original chest X-ray image. For fundus images, although the pixel loss during recovery exceeds 15 and approaches 32 (i.e., the first 5 bits change), this change has minimal impact on the overall image. Specifically, the least significant bits of the combined fundus image have changed, and the pixel value difference compared to the original image is only 1–2 points. Such a small change typically does not interfere with the doctor’s judgment in medical diagnosis, meaning that embedding the steganographic information does not affect clinical use.

**Table 7:** Quality evaluation metrics for hiding and recovery on different medical imaging datasets.

Dataset	Container vs. carrier			Embedded information	Reconstructed vs. original			
	PSNR	MSSIM	MSE		PSNR	MSSIM	MSE	MPE
Chest X-ray	38.82	0.972	35.70	Secret	39.34	0.985	14.32	7
	40.12	0.989	12.44	Secret code	35.73	0.939	102.3	13
Fundus imaging	39.58	0.981	27.84	Secret	37.55	0.964	19.89	11
	41.34	0.992	12.78	Secret code	29.36	0.914	225.9	28

These numerical values reflect the quality at the intermediate lossy recovery stage—prior to the application of cyclic code correction—rather than the quality of the final recovered image. This lossy recovery constitutes an intermediate step within the proposed dual-carrier mechanism. Specifically, the PSNR values of 39.34 dB for the recovered chest X-ray secret image and 35.73 dB for the recovered chest X-ray encoded image represent the quality of the network-extracted images before pixel-shift correction is applied. Following cyclic code correction, the pixel discrepancies for both the chest X-ray and fundus images are reduced to merely 1–2 intensity levels, which serves as the true metric for assessing recovery quality in steganography. The embedded payload information is indeed accurately and completely recoverable through the correction step—this is the core objective of the pixel-shift-based dual-carrier design.

## 6 Conclusion

The lossless medical privacy protection algorithm based on cyclic codes proposed in this paper successfully protects the privacy information of medical images while preserving the diagnostic quality and reliability of the images. By embedding the medical images and the encoded matrices into different carrier images, container images with virtually no visual difference are generated, ensuring the effective protection of privacy information. The recovery network can efficiently extract and reconstruct the encoded matrices and correct degraded medical images, recovering high-quality, lossless medical images. Experimental results demonstrate that the proposed algorithm achieves better performance than existing methods in medical image privacy protection and lossless recovery. Specifically, it achieves container PSNR of 40.08 dB and MSSIM of 0.988, confirming near-perceptual-indistinguishability; bit-level lossless recovery on the Brain Tumor and Chest X-ray datasets through the pixel-shift correction mechanism; and robustness against moderate noise and compression attacks while maintaining lossless recovery capability. Compared with existing deep learning-based steganography methods that recover only lossy approximations, and traditional RDH methods that achieve reversibility only under noise-free conditions, the proposed method provides a unique combination of lossless recovery and robustness.

It not only effectively prevents the leakage of sensitive information but also ensures the diagnostic quality of the images. Furthermore, the algorithm demonstrates strong robustness against noise and compression attacks that may occur during transmission, further enhancing the reliability and security of medical images in various application scenarios.

Additionally, the algorithm performs well on different datasets. Lossless recovery was achieved on chest X-ray images, and while there was some error on fundus images, the pixel loss was only 1–2 points, which has minimal impact on diagnosis.

Several promising directions for future research are identified. First, robustness against high-intensity attacks will be enhanced through stronger error-correcting codes, such as BCH or Reed-Solomon codes, and adversarial training. Second, the feasibility of a single-carrier lossless mechanism will be investigated via more sophisticated pixel-shift encoding or multi-level embedding strategies. Third, the current method, which operates on grayscale images, will be extended to color medical images by incorporating channel-wise cyclic correction and cross-channel attention mechanisms. Fourth, integration with lightweight encryption, such as selective ROI encryption, will provide dual-layer privacy protection against both steganalysis and brute-force attacks. Finally, clinical validation will be pursued in collaboration with medical institutions, including reader studies with radiologists to assess the method’s impact on diagnostic accuracy.

## Data Availability

Publicly available datasets were analyzed in this study. This data can be found here: https://www.kaggle.com/code/alexmas0n/brain-tumor-calssification; https://www.kaggle.com/datasets/paultimothymooney/chest-xray-pneumonia; https://www.kaggle.com/datasets/sabari50312/fundus-pytorch.
